# Structural, Electrical, Magnetic and Resistive Switching Properties of the Multiferroic/Ferroelectric Bilayer Thin Films

**DOI:** 10.3390/ma10111327

**Published:** 2017-11-20

**Authors:** Ming-Cheng Kao, Hone-Zern Chen, San-Lin Young, Kai-Huang Chen, Jung-Lung Chiang, Jen-Bin Shi

**Affiliations:** 1 Department of Electronic Engineering, Hsiuping University of Science and Technology, Taichung 41280, Taiwan; slyoung@hust.edu.tw; 2 Department of Electrical Engineering and Computer Science, Tung Fang Design Institute, Kaohsiung 82941, Taiwan; d9131802@gmail.com; 3 Department of Mobile Technology, Toko University, Chiayi 61363, Taiwan; cjunglung@gmail.com; 4 Department of Electronic Engineering, Feng Chia University, Taichung 40724, Taiwan; jbshi@fcu.edu.tw

**Keywords:** ferroelectric properties, multiferroic, magnetic properties, RRAM

## Abstract

Bi_0.8_Pr_0.2_Fe_0.95_Mn_0.05_O_3_/Bi_3.96_Gd_0.04_Ti_2.95_W_0.05_O_12_ (BPFMO/BGTWO) bilayer thin films with Multiferroic/Ferroelectric (MF/FE) structures were deposited onto Pt(111)/Ti/SiO_2_/Si(100) substrates by using the sol-gel method with rapid thermal annealing. The BPFMO/BGTWO thin films exhibited well-saturated ferromagnetic and ferroelectric hysteresis loops because of the electro-magnetic coupling induced by the MF/FE structure. The remnant magnetization (2Mr) and remnant polarization (2Pr) were 4.6 emu/cm^3^ and 62 μC/cm^2^, respectively. Moreover, the bipolar *I-V* switching curves of BPFMO/BGTWO bilayer thin films resistive random access memory (RRAM) devices were discussed, and investigated for LRS/HRS.

## 1. Introduction

Multiferroic/Ferroelectric (MF/FE) composite thin films have attracted considerable attention owing to their unique and exciting properties, which result from their combination of both ferroelectric and ferromagnetic materials [1,2,3,4,5]. These composite materials exhibit novel properties, such as electro-optic and electro-magnetic coupling, and therefore show great potential for use in many applications, including data storage, sensors, actuators, transducers and resistive random-access memory (RRAM) [6,7,8]. Multiferroic materials are well-known to simultaneously possess ferromagnetism and ferroelectricity, thus exhibiting spontaneous polarization and magnetization that can be reoriented by electric and magnetic fields, respectively [9,10,11,12]. An MF BiFeO_3_ (BFO) thin film with a rhombohedrally distorted perovskite structure has attracted much attention because it has a high FE Curie temperature of ~1103 K and an antiferromagnetic Néel temperature of ~643 K [13,14,15,16,17,18,19,20,21]. FE Bi_4_Ti_3_O_12_ (BTO) thin films are promising candidates for use in FeRAM applications owing to their large remnant polarization and good fatigue-free properties. Recently, lanthanide-substituted Bi_4_Ti_3_O_12_ (BTO), (Bi_4−x_Ln_x_)Ti_3_O_12_ (Ln = Gd, Nd, Sm and Pr) has been reported to show improved FE and fatigue resistance properties [22,23,24].

In this study, we report the fabrication of MF/FE bilayer thin films composed of Bi_0.8_Pr_0.2_Fe_0.95_Mn_0.05_O_3_ and Bi_3.96_Gd_0.04_Ti_2.95_W_0.05_O_12_ (BPFMO/BGTWO) structures and the fabrication of MF/FE-based RRAM. The BPFMO/BGTWO thin films were deposited on Pt(111)/Ti/SiO_2_/Si(100) substrates by the sol-gel spin-coating method with rapid thermal annealing in an oxygen atmosphere. The crystallization and microstructures of the thin films were determined by X-ray diffraction (XRD) patterns using a Rigaku D/max 2200 X-ray diffractometer with Cu-Kα radiation source, and scanning electron microscopy (SEM), respectively. Measurements of the FE hysteresis loops were performed using a Sawyer-Tower circuit. The magnetic hysteresis loops of the samples were analyzed at ambient temperature using a vibrating sample magnetometer (VSM). Additionally, the bipolar switching properties of the Bi_0.8_Pr_0.2_Fe_0.95_Mn_0.05_O_3_/Bi_3.96_Gd_0.04_Ti_2.95_W_0.05_O_12_ (BPFMO/BGTWO) bilayer thin film RRAM devices were discussed and investigated for low resistive state (LRS) and high resistive state (HRS).

## 2. Material and Methods

The general chemical formula of the precursor solution of Bi_0.8_Pr_0.2_Fe_0.95_Mn_0.05_O_3_ was prepared by the sol-gel method. Bismuth acetate (Bi(OOCCH_3_)_3_, Alfa, 99.99%+ purity), praseodymium acetate (Pr(OOCCH_3_)_3_·xH_2_O, Alfa (Haverhill, MA, USA), 99.9%+ purity), manganese acetate (Mn(C_2_H_3_O_2_)_2_·4H_2_O) and iron acetylacetonate (Fe(CH_3_COCHCOCH_3_)_3_, Alfa, 99.9%+ purity) were used as source materials. Propionic acid and 2-methoxyethanol was used as solvents. The purity of Bi(OOCCH_3_)_3_ (0.810852 g) and Pr(OOCCH_3_)_3_·xH_2_O (0.15902 g) were determined gravimetrically, then they were dissolved in propionic acid and 2-methoxyethanol to obtain sol compositions. The solutions were refluxed at 60 °C for 0.5 h under one atmosphere pressure. After the addition of Fe(CH_3_COCHCOCH_3_)_3_ (0.838779 g) and Mn(C_2_H_3_O_2_)_2_·4H_2_O (0.33513 g), the solutions of volume 10 mL were refluxed at 80 °C for 2 h to promote solution homogeneity. A stock solution of ~1 M concentration was obtained. A solution containing Bi_3.96_Gd_0.04_Ti_2.95_W_0.05_O_12_ was obtained using bismuth acetate (Bi(OOCCH_3_)_3_, Alfa, 99.99%+ purity), gadolinium nitrate hydrate (Gd(NO_3_)_3_·xH_2_O, Aldrich (St. Louis, MO, USA), 99.9%+ purity), tungsten isopropoxide (W[OCH(CH_3_)_2_]_6_, Aldrich, 99.9%+ purity) and titanium diisopropoxide bis(2,4-pentanedionate) (TIAA, Ti(OC_3_H_7_)_2_(CH_3_COCHCOCH_3_)_2_, Alfa, 99.9%+ purity). 2-methoxyethanol (CH_3_OC_2_H_4_OH) was used as a solvent. The purity of Bi(OOCCH_3_)_3_ (4.0137174 g) and Gd(NO_3_)_3_·xH_2_O (0.046039 g) were determined gravimetrically, then they were dissolved in 2-methoxyethanol in a 1:5 molar ratio of (Bi + Gd) to diol. The mixture was refluxed at 80 °C for 0.5 h under one atmospheric pressure. After adding TIAA (2.1256225 g) and W[OCH(CH_3_)_2_]_6_ (0.067295 g), the solution of volume 10 mL was refluxed at 80 °C for 2 h to promote homogeneity, and then cooled down to ambient temperature. Inductively coupled plasma mass spectrometry (ICP-MS) was used to confirm that the deviation from stoichiometry was within ±1%.

The BGTWO solution was spin-cast onto Pt/Ti/SiO_2_/Si(100) substrates at a spin rate of 2500 rpm for 30 s. After 15 times the spin-coated thin films, each coating step, the gel films were pyrolyzed on a hot plate at 300 °C for 2 min before final annealing. After multi-coating, the BSTTO thin films were annealed at 650 °C for 2 min using rapid thermal annealing (RTA) at a heating rate of 500 °C/min in the oxygen atmosphere. The desired thin film thickness of approximately 0.2 μm was achieved by repeating the spin-coating and annealing cycles. The BPFMO solution was then spin-cast 6 times onto the BGTWO/Pt/Ti/SiO_2_/Si(100) substrates using the same process as that described for BGTWO. Finally, the BPFMO/BGTWO thin films were annealed at 450 °C for 2 min by RTA at a heating rate of 400 °C/min in the oxygen atmosphere.

The crystallization and microstructures of the BGTWO and BPFMO/BGTWO thin films were analyzed using XRD and SEM, respectively. The top electrodes, with an area of 7.85 × 10^−3^ cm^2^, were prepared by DC sputtering of platinum through a mask onto the surface of the films. Measurements of the FE hysteresis loops and the leakage current were performed using a Sawyer-Tower circuit in a metal-ferroelectric-metal (MFM) configuration, respectively. The bipolar switching properties of RRAM devices are obtained by Agilent B1500 semiconductor parameter analyzer.

## 3. Results and Discussion

**Figure 1:**
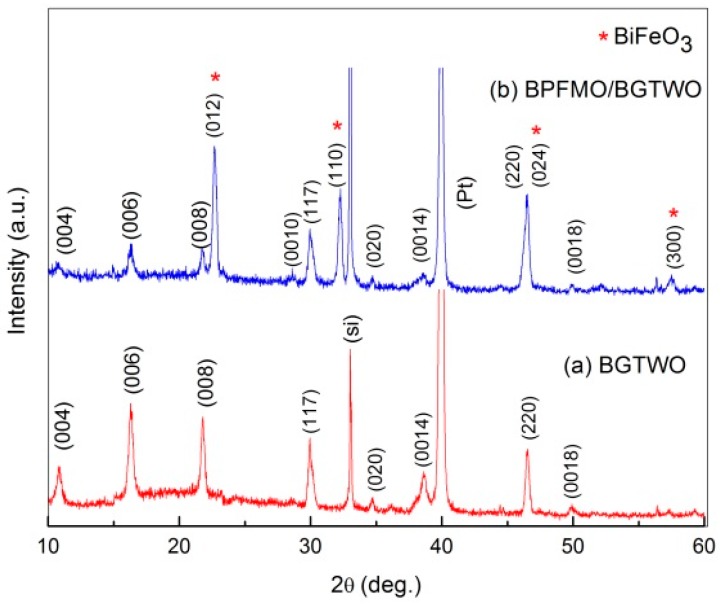
XRD spectra of (**a**) a pure BGTWO film and (**b**) a BPFMO/BGTWO bilayer thin film.

The XRD spectra of the pure BGTWO and BPFMO/BGTWO bilayer thin films are shown in Figure 1. The XRD peaks observed were similar to those of Bi_4_Ti_3_O_12_ and BiFeO_3_ (JCPDS cards). The BGTWO thin film showed the preferred (117) orientation of crystalline growth. Bismuth titanate crystals exhibit spontaneous polarizations (2Pr) of 4 and 50 μC/cm^2^, and coercive fields (2Ec) of 120 and 100 kV/cm along the *c*- and *a*-axis, respectively. Therefore, the degree of *a*-axis orientation is important in obtaining a large Pr. In the XRD pattern, the (117) diffraction peak is the most closely related to the *a*-axis orientation. The (006) diffraction peak is closely related to the *c*-axis orientation. It is clear that the BPFMO/BGTWO bilayer thin films enhance the intensity of the (006)-axis orientation of BGTWO, and the (012)-axis orientation of BPFMO, respectively.

**Figure 2:**
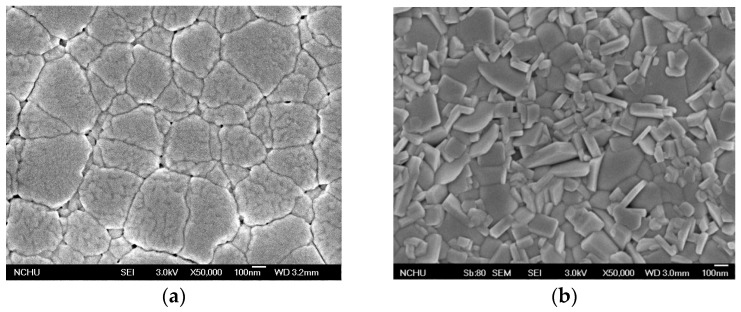

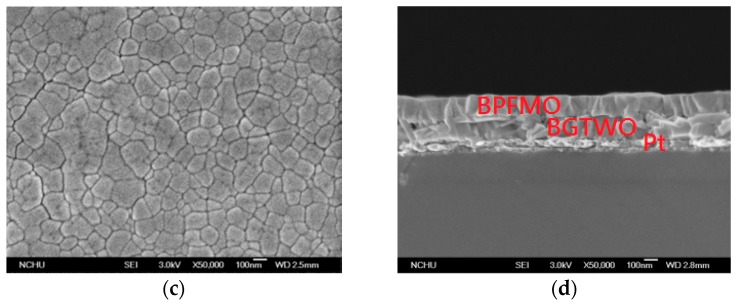
SEM topography image of (**a**) a pure BPFMO thin film; (**b**) a pure BGTWO thin film; (**c**) a BPFMO/BGTWO bilayer thin film; and (**d**) a typical cross section of a BPFMO/BGTWO bilayer thin film.

The surface microstructure of the BPFMO, BGTWO and BPFMO/BGTWO bilayer films, and a cross section of the BPFMO/BGTWO film are shown in Figure 2. As shown in Figure 2a–c, a smooth surface and improved crystallization structures were obtained for BPFMO, BGTWO and BPFMO/BGTWO films on the Pt/Ti/SiO_2_/Si(100) substrates. The SEM images revealed that the grains of the BPFMO/BGTWO bilayer thin films annealed under oxygen were dense and clear. Furthermore, the grains of the BPFMO/BGTWO bilayer thin film were smaller than those of the pure BPFMO film. A distinct interface is evident between the BPFMO and BGTWO layers in the composite film (Figure 2d), with the thickness of each layer being ~200 nm.

**Figure 3:**
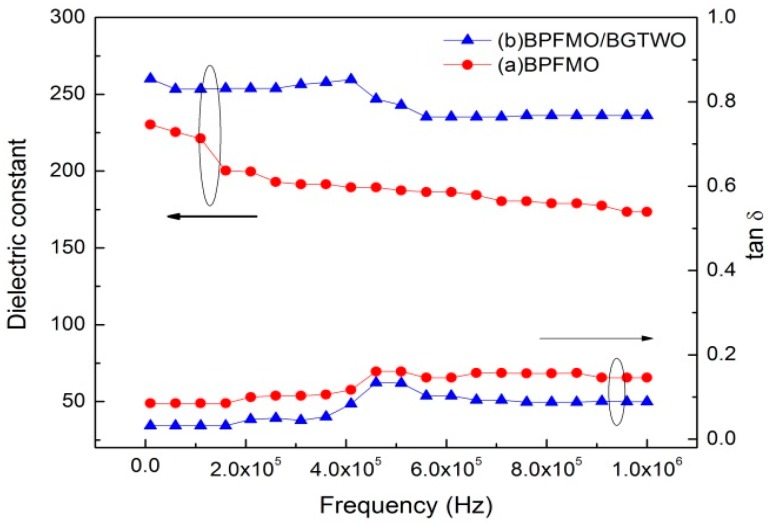
Dielectric constant (ε_r_) and tan δ of (**a**) a pure BPFMO film and (**b**) a BPFMO/BGTWO bilayer thin film.

The dielectric constant (ε_r_) and dielectric loss (tan δ) as functions of frequency for the BPFMO/BGTWO bilayer thin film at ambient temperature are shown in Figure 3. The ε_r_ at 400 kHz for the BPFMO/BGTWO bilayer thin film (250) was larger than that for the pure BPFMO thin film (180). The ε_r_ values of the BPFMO/BGTWO thin films decreased with an increase in frequency (Figure 3). It appears that the dielectric constant for the bilayer film was always greater than that of the pure film over the frequencies tested. There appeared to be a drop in value for the bilayer film between ~400 and 550 kHz, while the pure film exhibited a drop between ~80 and 180 kHz. It is plausible that this may occur because of the extrinsic resonance behavior induced by the microstructure deficiency of the thin films. The tan δ values of the bilayer films above 500 kHz exhibited a sharp increase that may be attributed to the switching of dipoles in the BPFMO/BGTWO thin films that cannot follow the applied high frequency electric field.

**Figure 4:**
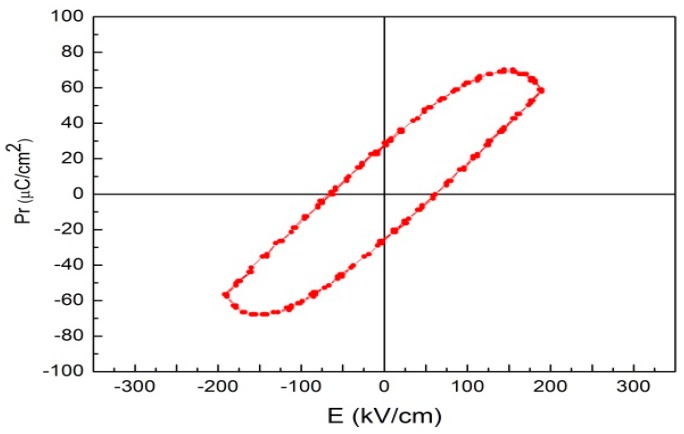
Ferroelectric hysteresis loops of a BPFMO/BGTWO bilayer thin film.

**Figure 5:**
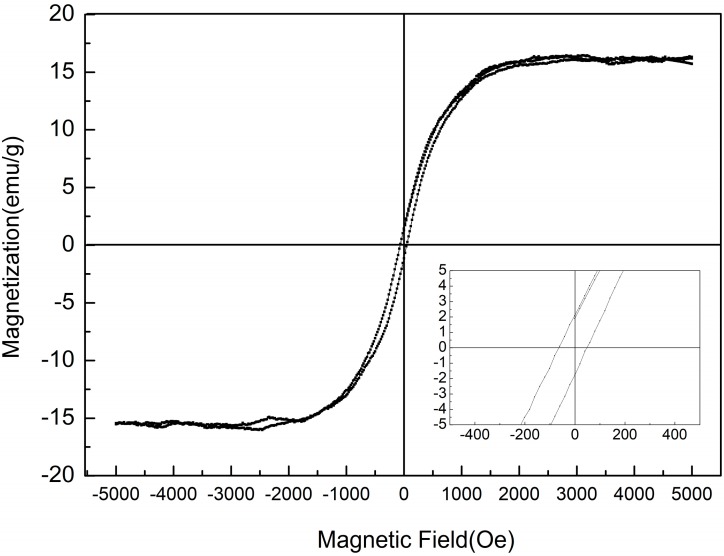
Magnetic hysteresis loop of a BPFMO/BGTWO bilayer thin film.

The FE hysteresis loop measured for the BPFMO/BGTWO thin film at different applied electric fields at ambient temperature is shown in Figure 4. The narrow loop observed was similar to that typically shown by an FE relaxor. Enhanced 2Pr (62 μC/cm^2^) and 2Ec (120 kV/cm) values were obtained for the BPFMO/BGTWO thin film. This may be the heterogeneous structure in the heat treatment, the atoms between the layers of mutual diffusion and traction, making the film within the oxygen vacancies reduced, resulting in enhanced ferroelectric properties. The magnetic hysteresis (M-H) loop of the BPFMO/BGTWO bilayer thin film, measured at ambient temperature with an applied magnetic field parallel to the plane of the samples, is shown in Figure 5. A well-developed M-H loop was observed from the BPFMO/BGTWO thin film, indicating the presence of an ordered magnetic structure. Doping Mn in the BFO thin film can effectively restrain the transition between Fe^3+^ and Fe^2+^, and can also reduce the oxygen vacancies. The Fe^2+^ ion production was due to oxygen vacancies, which would lead to an FM exchange interaction of Fe^2+^-O^2−^-Fe^3+^. In this work, the BPFMO/BGTWO heterostructure was able to reduce the antiferromagnetic Fe^2+^-O^2−^-Mn^3+^ interaction, and produced better magnetic properties. The ME coupling effect may be produced by a coupling of electric dipoles and electron spins through interfacial elastic strain transferred between ferroelectric and ferromagnetic components [25,26,27]. Additionally, the bilayer thin film exhibited a 2Mr of 4.6 emu/g and a saturation magnetization (Ms) of 15 emu/g. In this study, the BPFMO/BGTWO thin films exhibited coexisting electric and magnetic orders within a single multifunctional material, which indicates that the BPFMO/BGTWO thin films show great potential for a variety of applications.

**Figure 6:**
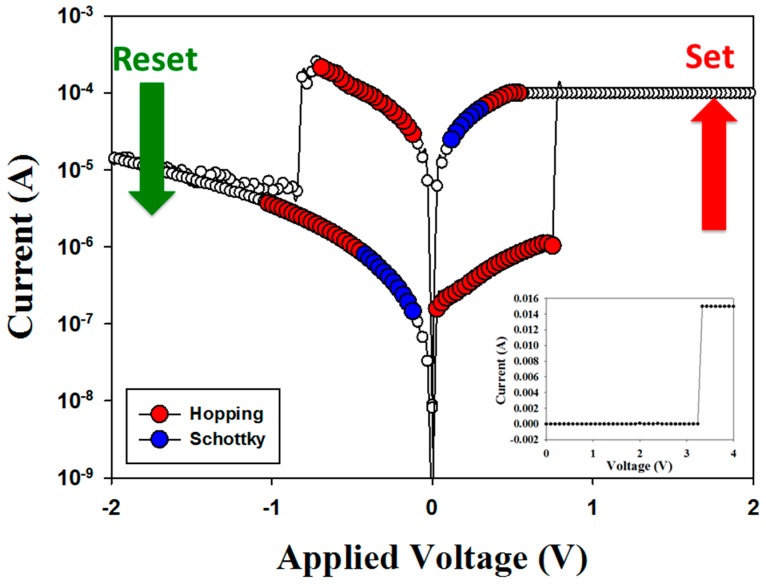
Bipolar switching properties of a BPFMO/BGTWO bilayer thin film.

Figure 6 presents the bipolar *I-V* switching curves of BPFMO/BGTWO bi-layer thin films for the typical 100 cycles. In the insert of Figure 6, a voltage of 3 V was applied to the initial forming process. The compliance current was 10 mA. Above the positive applied voltage for the RRAM devices, low current suddenly transferred to high current, which was denoted the set state. To define the reset state, the high current of the RRAM devices was transferred to low current.

In the set state, the electrical transferred conduction of the RRAM devices for low resistive status (LRS) exhibited the Schottky and hopping conduction mechanisms in *I-V* fitting curves. For HRS, the RRAM devices all exhibited the hopping conduction mechanism. Finally, the BPFMO/BGTWO bi-layer RRAM devices exhibited a different electrical conduction mechanism in the reset state. This electrical hopping conduction effect was caused by the electrons being transferred to the metal ion cluster for the multi-metallic filament forming process of the multiferroic/ferroelectric composite thin films.

## 4. Conclusions

High-quality multiferroic and ferroelectric BPFMO/BGTWO bilayer thin films were fabricated on Pt(111)/Ti/SiO_2_/Si(100) substrates. We demonstrated a convenient process for fabricating crack-free BPFMO/BGTWO bilayer thin films with a precise stoichiometric ratio following heat treatment. The leakage current density of the bilayer thin films decreased markedly. Additionally, the BPFMO/BGTWO bilayer thin films exhibited enhanced ferroelectric and ferromagnetic properties when compared with the pure BPFMO thin film, with a remnant polarization of 62 μC/cm^2^ and a remnant magnetization of 4.6 emu/g. Finally, the electrical conduction behaviors of BPFMO/BGTWO bilayer thin films film RRAM for set/reset state were proved, and exhibited the hopping conduction and Schottky emission mechanisms.
